# Correlation between LncRNA Profiles in the Blood Clot Formed on Nano-Scaled Implant Surfaces and Osseointegration

**DOI:** 10.3390/nano11030674

**Published:** 2021-03-09

**Authors:** Long Bai, Peiru Chen, Bin Tang, Ruiqiang Hang, Yin Xiao

**Affiliations:** 1 Key Laboratory for Ultrafine Materials of Ministry of Education, The State Key Laboratory of Bioreactor Engineering, East China University of Science and Technology, Shanghai 200237, China; bailong@ecust.edu.cn; 2 Laboratory of Biomaterial Surfaces & Interfaces, Institute of New Carbon Materials, Taiyuan University of Technology, Taiyuan 030000, China; tangbin@tyut.edu.cn; 3 Institute of Health and Biomedical Innovation, Queensland University of Technology, Brisbane 4059, Australia; 4 Australia-China Centre for Tissue Engineering and Regenerative Medicine, Queensland University of Technology, Brisbane 4059, Australia; 5 Beijing Proteome Research Center, State Key Laboratory of Proteomics, National Center for Protein Sciences (Beijing), Institute of Lifeomics, Beijing 102206, China; chenpeiru12@126.com

**Keywords:** implant, nano-scaled surface, blood clot, LncRNA, osseointegration, bone regeneration

## Abstract

Implant surfaces with a nanoscaled pattern can dominate the blood coagulation process resulting in a defined clot structure and its degradation behavior, which in turn influence cellular response and the early phase of osseointegration. Long non-coding (Lnc) RNAs are known to regulate many biological processes in the skeletal system; however, the link between the LncRNA derived from the cells within the clot and osseointegration has not been investigated to date. Hence, the sequence analysis of LncRNAs expressed within the clot formed on titania nanotube arrays (TNAs) with distinct nano-scaled diameters (TNA 15 of 15 nm, TNA 60 of 60 nm, TNA 120 of 120 nm) on titanium surfaces was profiled for the first time. LncRNA LOC103346307, LOC103352121, LOC108175175, LOC103348180, LOC108176660, and LOC108176465 were identified as the pivotal players in the early formed clot on the nano-scaled surfaces. Further bioinformatic prediction results were used to generate co-expression networks of LncRNAs and mRNAs. Gene Ontology and Kyoto Encyclopedia of Genes and Genomes pathway analyses revealed that distinct nano-scaled surfaces could regulate the biological functions of target mRNAs in the clot. LOC103346307, LOC108175175, and LOC108176660 upregulated mRNAs related to cell metabolism and Wnt, TGF-beta, and VEGF signaling pathways in TNA 15 compared with P-Ti, TNA 60, and TNA 120, respectively, whereas LOC103352121, LOC103348180, and LOC108176465 downregulated mRNAs related to bone resorption and inflammation through negatively regulating osteoclast differentiation, TNF, and NF-kappa signaling pathways. The results indicated that surface nano-scaled characteristics can significantly influence the clot-derived LncRNAs expression profile, which affects osseointegration through multiple signaling pathways of the targeted mRNAs, thus paving a way for better interpreting the link between the properties of a blood clot formed on the nano-surface and de novo bone formation.

## 1. Introduction

Osseointegration indicates a direct anchorage of a biomedical metal implant onto the host bone, allowing the newly formed bone to be attached directly to the surface of the implant [1]. Osseointegrated implants show promise to replace damaged joint tissues, alleviate pain, and restore bone function. However, implant loosening contributes to more than half of replacement failures due to the poor osseointegration between host bone and implant [2]. Fulfilled osseointegration requires rapid bone formation with a qualified volume, ensuring the incorporation and longevity of the implant [3]. The commercial pure titanium-based implant failed to satisfy this requirement due to its bioinert lacking bioactivity [4]. Surface modification in nano-scale on titanium-based implant has been verified to promote osseointegration [5,6]. However, the underlying mechanism pertaining to the nano-scaled surface-mediated osseointegration is not well-understood. Osseointegration is a sophisticated process that initiates immediately with clot formation on the implant surface. The blood clot has been recognized as a natural healing scaffold consisting of fibrin fiber structure and myriad immune cells including T cells, B cells, macrophages, and neutrophils [7,8]. The clot exerts a pivotal role in the manipulation of osseointegration, as it can modulate the early immune response and osteogenesis/angiogenesis through osteoimmunomodulation [8]. Efforts have been conducted to demonstrate the role of mRNAs within the clot on osseointegration in our recent study [8]. However, more is to be explored based on the recent advances in the potential role of the Long non-coding RNAs (LncRNAs) on cell differentiation, especially these within the clot on the osseointegration.

LncRNAs, whose length of the transcripts ranges from 200 nt to 100 kb, are emerging pivotal factors in the regulation of gene transcription and thus affect various aspects of cellular homeostasis, including proliferation, survival, migration, and genomic stability [9]. Numerous studies have demonstrated that the expression profile of LncRNAs is related to tissue regeneration and disease development, thus the LncRNAs profiling study will help to unravel their underlying functions [10]. However, the role of LncRNAs in the skeletal system and the regulation of osseointegration remain largely unclear to date. Specifically, no report was made on the expression profiles of LncRNAs in the early bone healing clot. LncRNA is multifaceted and varies differently from its locations, binding sites, and acting modes when exerting its biological function [11]. The regulating role of LncRNAs is not solitary but instead occurs through a large complex network that involves mRNAs, miRNAs, and proteins [12]. Based on our previous study [8], we demonstrated the nano-scaled surfaces could significantly influence the osteoimmunological reaction, angiogenesis, and osseointegration. In this study, we further investigated the expression of LncRNAs in blood clot formed on different nano-scaled surfaces and their potential impact on the osseointegration.

The nanostructured titania nanotube array (TNA) was chosen as the nano-scaled surfaces on the titanium implant due to the advantageous features of proven biocompatibility, thermal stability, and corrosion resistance [13]. Previous studies have demonstrated that surface modification of TNAs conferred the pristine titanium implant with enhanced osteogenesis, angiogenesis, and potentially induced favorable osseointegration [13,14]. However, osseointegration is a sophisticated process that involved multiple cells as aforementioned and the inconsistency of relevant in vitro/vivo studies requires a deeper investigation and clarification. Herein, TNAs with three distinct diameters (15, 60, and 120 nm) were fabricated aiming to unravel whether the distinct nano-surfaces can influence the LncRNAs profiles within the clot. Moreover, we specifically focused on the potential effect of the LncRNAs expression on the early phases of osseointegration, that is, whether the different LncRNAs profiles can a distinct osteoimmunomodulation effect on confer the nano-surfaces.

## 2. Materials and Methods

### 2.1. Surface Modification and Characterization of the Ti Implant

Ti implants shaped in rod (99.6% purity, the length of 5.0 mm, the diameter of 3.0 mm) were introduced for the in vivo study. The implants were ultrasonically cleaned in acetone, ethanol, and ultrapure water sequentially before the anodization. The titania nanotube arrays with different diameters on the surfaces were fabricated via an electrochemical cell (IT6120, ITECH, Shanghai, China) with a two-electrode configuration. The implants were used as the anode electrode while the platinum foil was set as the counter electrode. Electrochemical treatments were carried out in ethylene glycol solution containing 0.5 wt% ammonium fluoride (NH_4_F), 5 vol% methanol, and 5 vol% distilled water. The applied potentials were 5 V for 2 h, 30 V for 1 h, and 60 V for 10 min separately at room temperature. Afterwards, the as-prepared implants with distinct nano-scaled surfaces were ultrasonic-cleaned in ethanol for 10 min and air-dried. The surface morphology of the implants was characterized by a field-emission scanning electron microscope (SEM, JSM-7001F, Tokyo, Japan).

### 2.2. In Vitro Observation of the Platelet Activation on the Nano-Scaled Surfaces

Blood was collected in 3.8% sodium citrate (9:1, *v*/*v*) from healthy aspirin-free donors following informed consent at the Australian Red Cross Blood Bank. All procedures were carried out under approval by the University Human Research Ethics Committee at the Queensland University of Technology (1500000918). Citrated blood was centrifuged at 1200 rpm for 10 min and the platelet-rich plasma (PRP) was collected using the 1.5 mL EP tubes. To investigate the platelet activation, the implants were placed in a 96-well plate (Corning, St. Louis, MO, USA) and 50 μL PRP was dropped on each specimen. The plate was incubated at 37 °C for 30 min. Then the activation of the platelets on the implants was observed by SEM after gradient elution via ethanol. 

### 2.3. In Vivo Clot Observation on the Nano-Scaled Surfaces

The animal surgery was done consistent with protocols approved by the Animal Research Committee of Taiyuan University of Technology (TYUT202001003). A total of 8 New Zealand rabbits (male, 9 to 10 months) were used. The animals were sedated with 2 mg/kg intramuscular midazolam, and general anesthesia was conducted with an intramuscular injection of 50 mg/kg ketamine and 15 mg/kg xylazine. With animals under local anesthesia with oxybuprocaine 0.4%, four cylindrical titanium implants covered with nanotubes were placed on each distal surface of the bilateral femoral condyles of an animal. After 24 h of implantation, the animals were euthanized, and the implants were harvested immediately and then fixed in 4% PFA. The clot morphology was observed by SEM after the process with the standard procedures.

### 2.4. Histological Analysis

After 8 weeks of implantation, the implants were dehydrated and embedded in the resin. EXAKT saw was introduced to process the specimens into several sections with 200 μm in thickness. Afterwards, 50 μm sections were grinded then polished through the EXAKT grinder (EXAKT 400 CS, Norderstedt, Germany). The sections were then stained with Toluidine blue. Three histological sections were evaluated via the Leica microscope and quantified by Image J.

### 2.5. LncRNAs Profile of the Clot on the Nano-Scaled Surfaces

Three days after implantation, the other 4 animals were euthanized, and the clot was collected from the surface of the specimens for total RNA extraction using Trizol (Invitrogen). Subsequently, total RNA was qualified and quantified using a NanoDrop and Agilent 2100 bioanalyzer (Thermo Fisher Scientific, MA, USA). For the Lnc-RNA profile detection, ribosomal RNA (rRNA) was removed using target-specific oligos and RNase H reagents were used to deplete both cytoplasmic (5S rRNA, 5.8S rRNA, 18S rRNA, and 28S rRNA) and mitochondrial ribosomal RNA (12S rRNA and 16S rRNA) from total RNA preparations. Following SPRI beads purification, the RNA was fragmented into small pieces using divalent cations under elevated temperature. The cleaved RNA fragments were copied into the first-strand cDNA using reverse transcriptase and random primers, followed by second-strand cDNA synthesis using DNA Polymerase I and RNase H. This process would remove the RNA template and synthesizes a replacement strand, incorporating dUTP in place of dTTP to generate ds cDNA. These cDNA fragments then had the addition of a single ‘A’ base and subsequent ligation of the adapter. After UDG treatment, the incorporation of dUTP quenched the second strand during amplification. The products were enriched with PCR to create the final cDNA library. The libraries were assessed quality and quantity using two methods: check the distribution of the size of the fragments using the Agilent 2100 bioanalyzer, and quantify the library using real-time quantitative PCR (QPCR) (TaqMan Probe, St. Louis, MO, USA). The qualified libraries were sequenced pair end on the BGISEQ-500 System. The sequencing platform of BGI-500 (BGI, Shenzhen, China) was used to obtain the LncRNA gene expression profiles. Quality control checks were performed to confirm sequencing saturation and gene mapping distribution. Fragments per Kilobase of transcript per Million mapped reads (FPKM) value were used to express relative gene abundance. Different LncRNAs in comparison with the nanotubes with a diameter of 15 nm were analyzed. RNAplex was used to reveal the potential targeted mRNAs of the LncRNAs. The target mRNAs were then subjected to enrichment analysis of GO functions and KEGG pathways.

### 2.6. Statistical Analysis

The quantitative data were displayed as means ± standard deviation (SD). The data were statistically analyzed using the software SPSS. Statistical analysis was performed using one-way ANOVA methodology. Significance and high significance were indicated at *p* values < 0.05 and 0.01, respectively.

## 3. Results and Discussion

**Figure 1:**
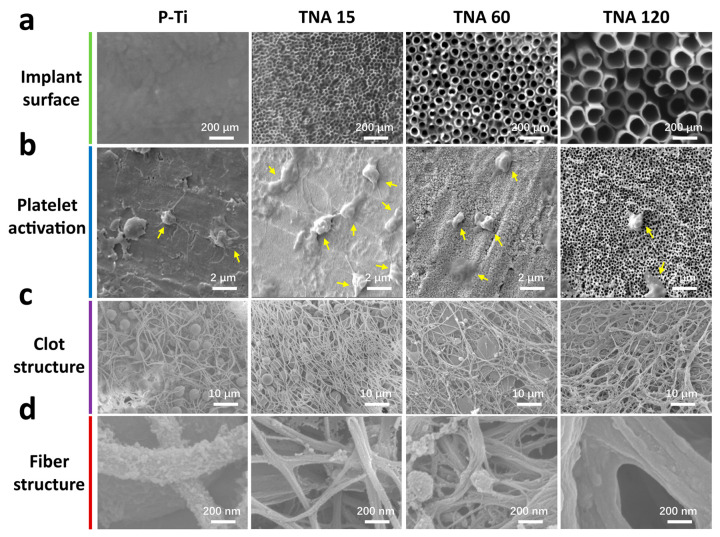
(**a**) Scanning electron microscope (SEM) images of the surface of P-Ti and TNAs. (**b**) Platelet activation (yellow arrows) on the surfaces. (**c**,**d**) Clot and fiber structures on the surfaces.

The surface morphology of P-Ti and TNAs is shown in Figure 1a. Highly ordered TNAs with distinct diameters are obtained after one-step anodization. The average diameter of TNAs is 15, 60, and 120 nm, thus are denoted as TNA 15, TNA 60, and TNA 120 respectively.

Figure 1b displays the platelet morphology adhered to the surfaces of the implants after 30 min of incubation. TNA 15 attracts more platelet adhesion and enables a significant activation morphology manifested by a huge extension area with abundant lamellipodia and filopodia in comparison with that on other groups.

After implanted for 3 days, the clot morphology on the surfaces of the implants is shown in Figure 1c and detailed information is displayed in Figure 1d. Clots with a much more compact and thinner fiber network formed on TNA 15, while a structure with larger pores with thicker fibers was observed on other groups, especially on TNA 120.

**Figure 2:**
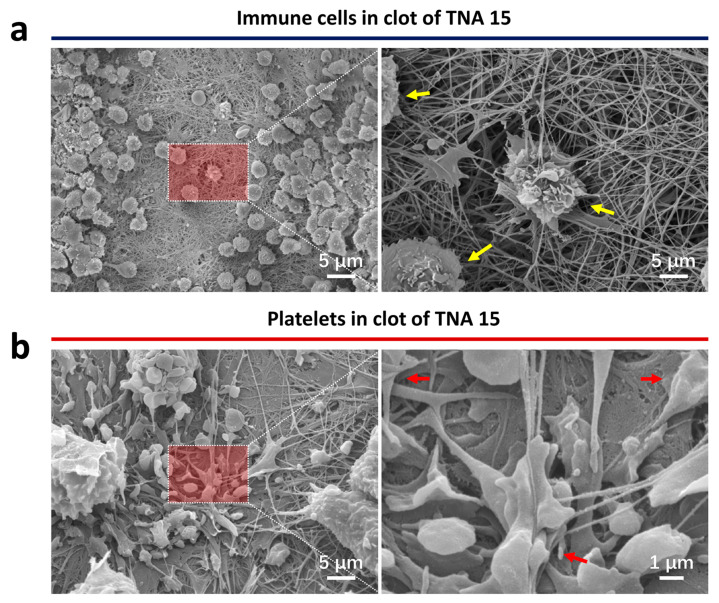
(**a**) SEM images of the immune cell (yellow arrows) morphology within the clot on the specimens. (**b**) SEM images of the platelet activations (red arrows) within the clot on the specimens. The right image is magnified from the left one.

Figure 2 discloses the detailed information of the in vivo clot features on TNA 15 after 24 h of implantation. Abundant activated immune cells can be observed within the clot (Figure 2a); meanwhile, numerous platelets with a huge degree of activation are also seen near the immune cells (Figure 2b). These results are consistent with the aforementioned in vitro outcome that TNA 15 enables a significant activation of immune cells and platelet activity [8].

**Figure 3:**
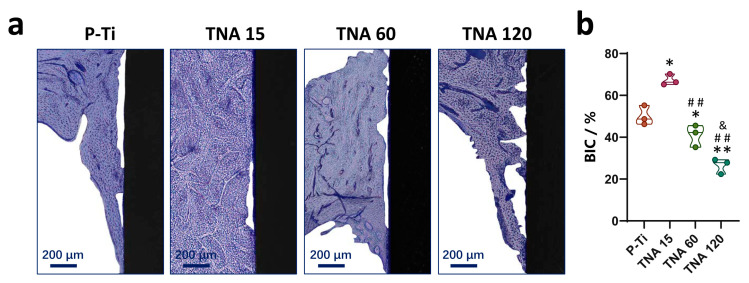
(**a**) Representative histological images of peri-implant bone tissue. (**b**) Quantitative results of BIC. * *p* < 0.05 compared to P-Ti, ^&^ *p* < 0.05 compared to TNA 60, ** *p* < 0.01 compared to P-Ti, ^##^ *p* < 0.01 compared to TNA 15.

In vivo osseointegration results of the modified implant are shown in Figure 3. Figure 3a displays the representative images of the bone-to-implant interface and peri-implant bone tissue stained with the toluidine blue. Similarly, significantly elevated BIC can be observed near the surface of TNA 15 from the images and further verified via the quantitative results in Figure 3b while compared with that of other groups [8]. Accordingly, TNA 15 was chosen as a reference group in the following study to investigate the regulation effect of the LncRNA profile within the clot.

**Figure 4:**
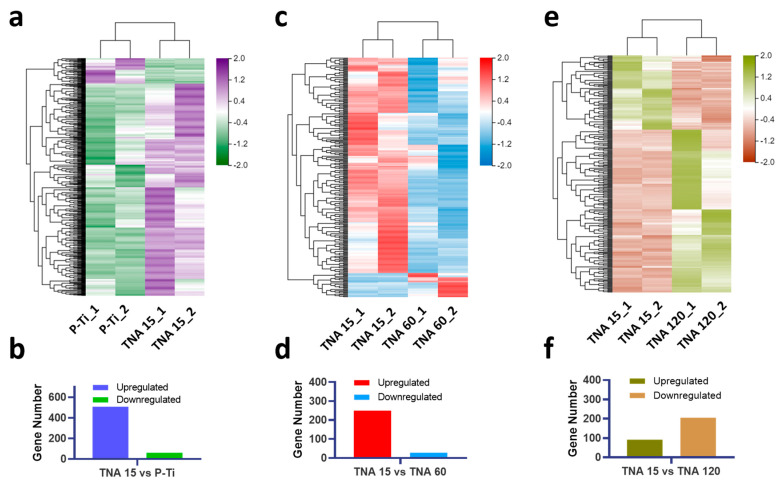
Visualization of different LncRNA expression profiles with heatmap. (**a**) P-Ti vs. TNA 15. (**b**) TNA 60 vs. TNA 15. (**c**) TNA 120 vs. TNA 15. Quantitative results of the LncRNA expression profiles. (**d**) P-Ti vs. TNA 15. (**e**) TNA 60 vs. TNA 15. (**f**) TNA 120 vs. TNA 15.

Figure 4 depicts the different LncRNAs expression profiles among groups. A total of 508 LncRNAs are significantly upregulated and 61 LncRNAs are downregulated of TNA 15 when compared with P-Ti (Figure 4a,b). 250 LncRNAs are significantly upregulated and 28 LncRNAs are downregulated of TNA 60 when compared with TNA 15 (Figure 4c,d). Similarly, 92 LncRNAs are significantly upregulated and 205 LncRNAs are downregulated of TNA 120 when compared with TNA 15 (Figure 4e,f).

**Figure 5:**
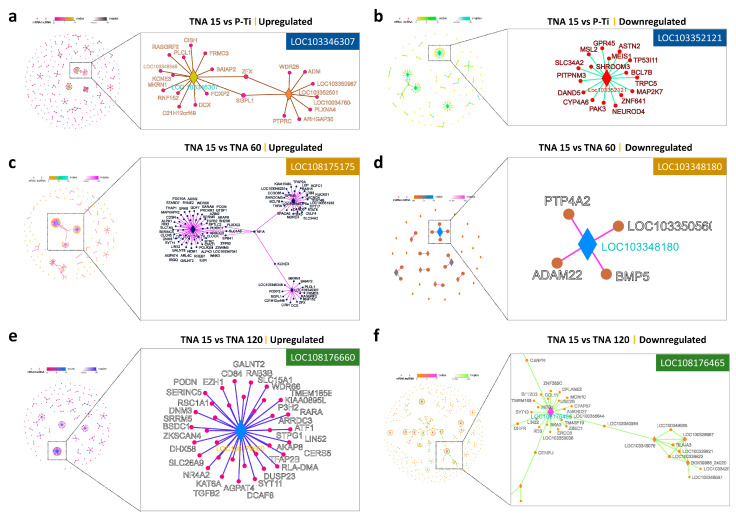
(**a**) Key LncRNA identified within the upregulated profile in TNA 15 vs. P-Ti. (**b**) Key LncRNA identified within the downregulated profile in TNA 15 vs. P-Ti. (**c**) Key LncRNA identified within the upregulated profile in TNA 15 vs. TNA 60. (**d**) Key LncRNA identified within the downregulated profile in TNA 15 vs. TNA 60. (**e**) Key LncRNA identified within the upregulated profile in TNA 15 vs. TNA 120. (**f**) Key LncRNA identified within the downregulated profile in TNA 15 vs. TNA 120.

Figure 5 indicates key LncRNAs involved in the regulation of LncRNA-mRNA among groups. LOC103346307, LOC108175175, and LOC108176660 targeted most of the upregulated mRNAs in TNA 15 compared with P-Ti, TNA 60, and TNA 120, respectively, whereas LOC103352121, LOC103348180, and LOC108176465 targeted most of the downregulated mRNAs.

**Figure 6:**
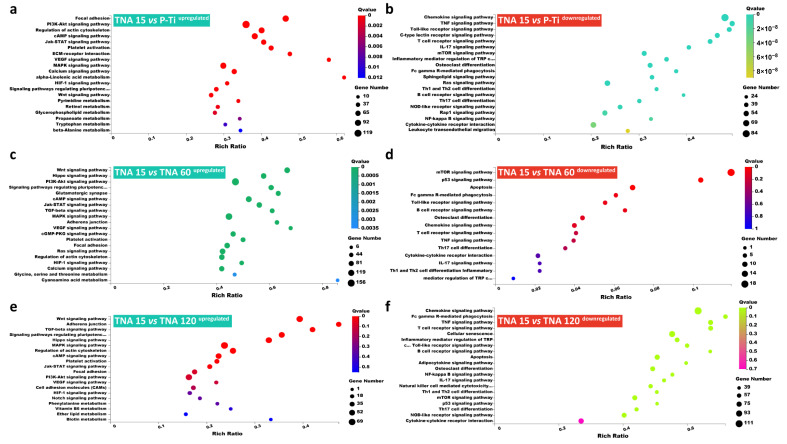
(**a**) KEGG pathway enrichment analysis of the upregulated LncRNAs targeted mRNAs within the comparison TNA 15 vs. P-Ti. (**b**) KEGG pathway enrichment analysis of the downregulated LncRNAs targeted mRNAs within the comparison TNA 15 vs. P-Ti. (**c**) KEGG pathway enrichment analysis of the upregulated LncRNAs targeted mRNAs within the comparison TNA 60 vs. P-Ti. (**d**) KEGG pathway enrichment analysis of the downregulated LncRNAs targeted mRNAs within the comparison TNA 60 vs. P-Ti. (**e**) KEGG pathway enrichment analysis of the upregulated LncRNAs targeted mRNAs within the comparison TNA 120 vs. P-Ti. (**f**) KEGG pathway enrichment analysis of the downregulated LncRNAs targeted mRNAs within the comparison TNA 120 vs. P-Ti.

**Figure 7:**
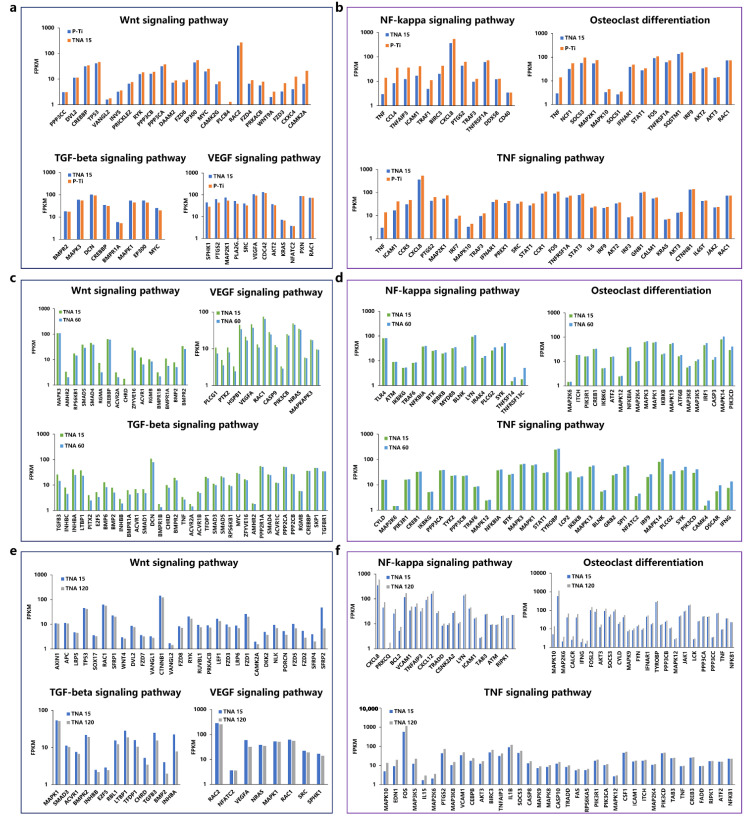
(**a**) Upregulated genes in the Wnt, TGF-beta, and VEGF signaling pathways within the comparison TNA 15 vs. P-Ti. (**b**) Downregulated genes in the osteoclast differentiation, TNF, NF-kappa signaling pathways within the comparison TNA 15 vs. P-Ti. (**c**) Upregulated genes in the WNT, TGF-beta, and VEGF signaling pathways within the comparison TNA 15 vs. TNA 60. (**d**) Downregulated genes in the osteoclast differentiation, TNF, NF-kappa signaling pathways within the comparison TNA 15 vs. TNA 60. (**e**) Upregulated genes in the Wnt, TGF-beta, and VEGF signaling pathways within the comparison TNA 15 vs. TNA 120. (**f**) Downregulated genes in the osteoclast differentiation, TNF, and NF-kappa signaling pathways within the comparison TNA 15 vs. TNA 120.

LncRNAs targeted mRNAs were introduced to KEGG pathway enrichment analysis (Figure 6) and the detailed gene expression in the pathways is shown in Figure 7. Figure 6a,b indicate that upregulated mRNAs in group TNA 15 vs. P-Ti were significantly enriched in growth metabolism-related signaling pathways such as the Focal adhesion, PI3K-Akt signaling pathway, Regulation of actin cytoskeleton, cAMP signaling pathway, Jak-STAT signaling pathway, and platelet activation, while the downregulated mRNAs are significantly enriched in inflammation-related signaling pathways such as the Chemokine signaling pathway, TNF signaling pathway, Toll-like receptor signaling pathway, C-type lectin receptor signaling pathway, T cell receptor signaling pathway, and IL-17 signaling pathway. Figure 6c,d indicate that upregulated mRNAs in group TNA 15 vs. TNA 60 were significantly enriched in growth metabolism-related signaling pathways such as the Wnt signaling pathway, Hippo signaling pathway, PI3K-Akt signaling pathway, Signaling pathways regulating pluripotency of stem cells, cAMP signaling pathway, and Jak-STAT signaling pathway, while the downregulated mRNAs were significantly enriched in inflammation-related signaling pathways such as the mTOR signaling pathway, p53 signaling pathway, Apoptosis, Fc gamma R-mediated phagocytosis, Toll-like receptor signaling pathway, and B cell receptor signaling pathway. Figure 6e,f indicate that upregulated mRNAs in group TNA 15 vs. TNA 60 were significantly enriched in growth metabolism-related signaling pathways such as the Wnt signaling pathway, Adherens junction, TGF-beta signaling pathway, Signaling pathways regulating pluripotency of stem cells, Hippo signaling pathway, and MAPK signaling pathway, while the downregulated mRNAs were significantly enriched in inflammation-related signaling pathways such as the Chemokine signaling pathway, Fc gamma R-mediated phagocytosis, T cell receptor signaling pathway, Cellular senescence, and Inflammatory mediator regulation of TRP.

Notably, WNT, TGF-beta, and VEGF signaling pathways had more synchronized appearances in TNA groups when compared with others. During osseointegration, Wnt signaling, which plays a pivotal role in MSC lineage commitment and progression, was implicated in proximal-distal outgrowth and dorsoventral limb patterning and, later, in osteogenesis mostly through the canonical pathway but also involving noncanonical elements [15]. Inhibition of WNT signaling resulted in low bone mass in osteoporosis-pseudoglioma syndrome [15]. Additionally, a total of 23 genes were significantly enhanced when compared with P-Ti. TGF-beta signaling is another crucial pathway evidenced to regulate bone mass and quality and loss of TGF-beta signaling also reduce bone matrix mineralization [16]. Besides the enhancement of de novo bone formation, angiogenesis at the bone-implant interface is recognized as a prerequisite for a fulfilled osseointegration [17]. VEGF signaling is capable of activating eNOS that is responsible for the production of vascular nitric oxide (NO), which consequently contributes to neovascularization [18]. Similarly, downregulation of osteoclast differentiation, TNF, and NF-kappa signaling pathways was identified in TNA groups when compared with others.

Osteoclasts are bone-resorbing multinucleated cells derived from hematopoietic precursors that are formed in the bone marrow through osteoclast differentiation of macrophages [19]. Osteoclasts break down de novo bone formation by secreting proteases, a process is known as bone resorption via degrading type I collagen and promote osteoclastogenesis [20]. The TNF signaling pathway functions in vivo to increase osteoclast precursors, as well as indirectly increasing osteoclastogenesis through augmentation of RANK expression on osteoclast precursors, plays a major role in promoting osteoclastogenesis and bone resorption [21]. The NF kappa signaling pathway is a family of transcription factors that have been comprehensively studied in the promotion of osteoclasts function [22]. Increasing evidence suggested that the pathway activation decreases osteogenic differentiation and suppresses de novo bone formation [23]. Additionally, the NF kappa signaling pathway plays an important regulatory role in the developmental process of inflammation in combination with its downstream inflammatory cytokines interleukin-6 (IL-6), interleukin-1β (IL-1β), and TNF-α, while suppression of inflammation is beneficial to osseointegration [24]. Accordingly, the LncRNAs identified herein contribute to the fulfilled osseointegration of TNA 15 through activation of Wnt, TGF-beta, and VEGF signaling pathways and the suppression of osteoclast differentiation, TNF, and NF-kappa signaling pathways.

**Figure 8:**
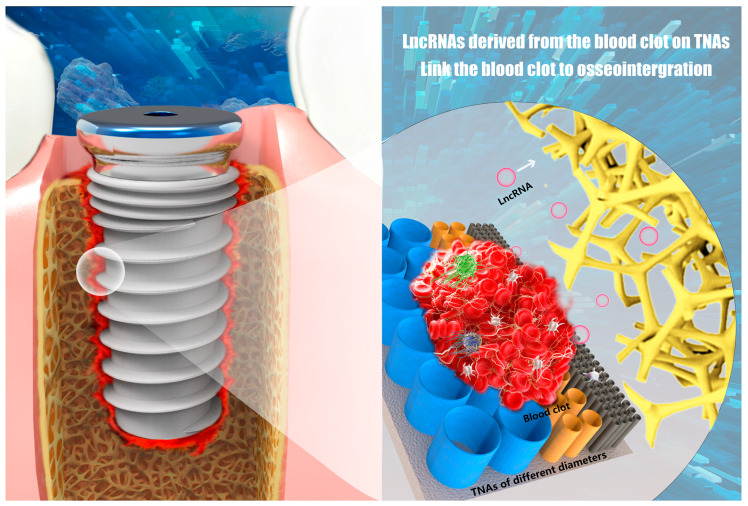
Illustration of the correlation between LncRNAs in the Blood Clot formed on Nano-Scaled Implant Surfaces and Osseointegration.

In our previous study, TNA 15 was shown to be highly promising for enhancing osseointegration through manipulating a fulfilled osteoimmune microenvironment by using the specialized thinner, porous blood clot fibrous network and the releasing of growth factors (PDGF-AB, TGF-beta) [8]. Herein, we depicted the results from a different viewpoint and deepened the current understanding of the osseointegration (Figure 8). Firstly, the study unprecedently indicated that TNA 15 can manipulate the expressions of LncRNAs within the clot, and thus highlighted that single nano-surfaces can significantly regulate LncRNAs profiles. Then, it was found that among hundreds of identified LncRNAs in each group, there are always several key LncRNAs that play a pivotal role in osteoimmunomodulation through regulation of multiple signaling pathways with their targeting mRNAs. Moreover, the study advanced the current comprehension of LncRNAs which have a powerful function in regulating de novo bone formation, and thus, provided a new paradigm for surface modification of the implantable materials to enhance osseointegration. The further study shall pay attention to deepen the understanding of the LncRNA modulated osteoimmune response during bone formation. Additionally, an in vitro study using larger size specimens is of interest to verify the in vivo effect of the blood clot-derived LncRNAs.

## 4. Conclusions

We demonstrated that distinct nano-surfaces are capable of regulating LncRNAs expression within the clot, and 6 key LncRNAs were identified in each comparison exerting a pivotal role in manipulating the expression of the targeted mRNA. In the TNA15 group, the LncRNAs targeted mRNAs subsequently manipulate a favorable osteogenesis microenvironment through upregulation of Wnt, TGF-beta, and VEGF signaling pathways and suppression of osteoclast differentiation, TNF, and NF-kappa signaling pathways, which resulted in promoted osseointegration. The findings firstly indicated the link of nano-surfaces on the LncRNAs expression in the blood clot and demonstrated that LncRNAs may strongly impact the osseointegration through the targeted mRNAs.

## Data Availability

The data presented in this study are available on request from the corresponding author.

## References

[1] Guglielmotti M.B., Olmedo D.G., Cabrini R.L. (2019). Research on implants and osseointegration. Periodontology 2000.

[2] Apostu D., Lucaciu O., Berce C., Lucaciu D., Cosma D. (2018). Current methods of preventing aseptic loosening and improving osseointegration of titanium implants in cementless total hip arthroplasty: A review. J. Int. Med. Res..

[3] Agarwal R., García A.J. (2015). Biomaterial strategies for engineering implants for enhanced osseointegration and bone repair. Adv. Drug Delivery Rev..

[4] Bosshardt D.D., Chappuis V., Buser D. (2017). Osseointegration of titanium, titanium alloy and zirconia dental implants: Current knowledge and open questions. Periodontology 2000.

[5] Souza J.C.M., Sordi M.B., Kanazawa M., Ravindran S., Henriques B., Silva F.S., Aparicio C., Cooper L.F. (2019). Nano-scale modification of titanium implant surfaces to enhance osseointegration. Acta Biomater..

[6] Bai L., Hang R., Gao A., Zhang X., Huang X., Wang Y., Tang B., Zhao L., Chu P.K. (2015). Nanostructured titanium–silver coatings with good antibacterial activity and cytocompatibility fabricated by one-step magnetron sputtering. Appl. Surf. Sci..

[7] Shahneh F., Alexandra G., Klein M., Frauhammer F., Bopp T., Schäfer K., Raker V., Becker C. (2020). Specialized regulatory T cells control venous blood clot resolution through SPARC. Blood.

[8] Bai L., Zhao Y., Chen P., Zhang X., Huang X., Du Z., Crawford R., Yao X., Tang B., Hang R. (2020). Targeting Early Healing Phase with Titania Nanotube Arrays on Tunable Diameters to Accelerate Bone Regeneration and Osseointegration. Small.

[9] Wu Z., Liu X., Liu L., Deng H., Zhang J., Xu Q., Cen B., Ji A. (2014). Regulation of lncRNA expression. Cell. Mol. Biol. Lett..

[10] Wapinski O., Chang H.Y. (2011). Long noncoding RNAs and human disease. Trends Cell Biol..

[11] Fatica A., Bozzoni I. (2014). Long non-coding RNAs: New players in cell differentiation and development. Nat. Rev. Genet..

[12] Young R.S., Ponting C.P. (2013). Identification and function of long non-coding RNAs. Essays Biochem..

[13] Bai L., Wu R., Wang Y., Wang X., Zhang X., Huang X., Qin L., Hang R., Zhao L., Tang B. (2016). Osteogenic and angiogenic activities of silicon-incorporated TiO_2_ nanotube arrays. J. Mater. Chem. B.

[14] Bai L., Yang Y., Mendhi J., Du Z., Hao R., Hang R., Yao X., Huang N., Tang B., Xiao Y. (2018). The effects of TiO2 nanotube arrays with different diameters on macrophage/endothelial cell response and ex vivo hemocompatibility. J. Mater. Chem. B.

[15] Baron R., Kneissel M. (2013). WNT signaling in bone homeostasis and disease: From human mutations to treatments. Nat. Med..

[16] Dole N.S., Mazur C.M., Acevedo C., Lopez J.P., Monteiro D.A., Fowler T.W., Gludovatz B., Walsh F., Regan J.N., Messina S. (2017). Osteocyte-Intrinsic TGF-β Signaling Regulates Bone Quality through Perilacunar/Canalicular Remodeling. Cell Rep..

[17] Langen U.H., Pitulescu M.E., Kim J.M., Enriquez-Gasca R., Sivaraj K.K., Kusumbe A.P., Singh A., Di Russo J., Bixel M.G., Zhou B. (2017). Cell-matrix signals specify bone endothelial cells during developmental osteogenesis. Nat. Cell Biol..

[18] Ahmad S., Hewett P.W., Wang P., Al-Ani B., Cudmore M., Fujisawa T., Haigh J.J., le Noble F., Wang L., Mukhopadhyay D. (2006). Direct evidence for endothelial vascular endothelial growth factor receptor-1 function in nitric oxide–mediated angiogenesis. Circ. Res..

[19] Park J.H., Lee N.K., Lee S.Y. (2017). Current Understanding of RANK Signaling in Osteoclast Differentiation and Maturation. Mol. Cells.

[20] Boyle W.J., Simonet W.S., Lacey D.L. (2003). Osteoclast differentiation and activation. Nature.

[21] Zhao B. (2017). TNF and Bone Remodeling. Curr. Osteoporos. Rep..

[22] Chang J., Wang Z., Tang E., Fan Z., McCauley L., Franceschi R., Guan K., Krebsbach P.H., Wang C.-Y. (2009). Inhibition of osteoblastic bone formation by nuclear factor-κB. Nat. Med..

[23] Krum S.A., Chang J., Miranda-Carboni G., Wang C.-Y. (2010). Novel functions for NFκB: Inhibition of bone formation. Nat. Rev. Rheumatol..

[24] Bai L., Du Z., Du J., Yao W., Zhang J., Weng Z., Liu S., Zhao Y., Liu Y., Zhang X. (2018). A multifaceted coating on titanium dictates osteoimmunomodulation and osteo/angio-genesis towards ameliorative osseointegration. Biomaterials.

